# Sex disparities in cardiovascular health metrics among rural-dwelling older adults in China: a population-based study

**DOI:** 10.1186/s12877-021-02116-x

**Published:** 2021-03-04

**Authors:** Xiaolei Han, Ziying Jiang, Yuanjing Li, Yongxiang Wang, Yajun Liang, Yi Dong, Shi Tang, Yifeng Du, Chengxuan Qiu

**Affiliations:** 1 Department of Neurology, Shandong Provincial Hospital, Cheeloo College of Medicine, Shandong University, No. 324 Jingwuweiqi Road, Jinan, 250021 Shandong People’s Republic of China; 2 Aging Research Center and Center for Alzheimer Research, Department of Neurobiology, Care Sciences and Society, Karolinska Institutet-Stockholm University, Stockholm, Sweden; 3 Department of Neurology, Shandong Provincial Hospital affiliated to Shandong First Medical University, Jinan, Shandong People’s Republic of China; 4 Department of Global Public Health, Karolinska Institutet, Stockholm, Sweden

**Keywords:** Cardiovascular health metrics, Rural elderly, Sex, Population-based study

## Abstract

**Background:**

Cardiovascular health (CVH) metrics among Chinese older adults are poorly understood. We investigated sex disparities in CVH metrics and their management among rural-dwelling older adults in China.

**Methods:**

This community-based study included 5026 participants (age ≥ 65 years; 57.2% women) in the baseline survey of a multimodal intervention study in rural China. In March–September 2018, data were collected through face-to-face interviews, clinical examinations, and laboratory tests. We defined six CVH metrics (three behavioral factors—smoking, body mass index, and physical activity; three biological factors—blood pressure, total cholesterol, and blood glucose) following the modified American Heart Association’s recommendations. We performed descriptive analysis separately for men and women.

**Results:**

Of all participants, only 0.8% achieved ideal levels in all six CVH metrics. Men were more likely than women to have ideal levels in all CVH metrics but smoking. Women had higher prevalence of ideal global (9.7% vs. 7.8%) and behavioral (18.3% vs. 9.5%) CVH metrics (*p* < 0.001), whereas men had higher prevalence of ideal biological CVH metrics (5.4% vs. 3.5%, *p* < 0.001). The prevalence of ideal global and behavioral CVH metrics increased with age in both women and men (*p* for trend< 0.001). Women were more likely to be aware of their hypertension and diabetes, and to receive antihypertensive treatment, while men were more likely to achieve the goal of high cholesterol treatment (*p* < 0.05).

**Conclusions:**

The CVH metrics among older adults living in the rural communities in China are characterized by an extremely low proportion of optimal global CVH metrics and distinct sex differences, alongside poor management of major biological risk factors.

**Trial registration:**

ChiCTR1800017758 (Aug 13, 2018).

**Supplementary Information:**

The online version contains supplementary material available at 10.1186/s12877-021-02116-x.

## Background

Cardiovascular disease (CVD) is the leading cause of death in China, and the burden of CVD is projected to steadily increase in the coming decades, driven primarily by population aging as well as rapid economic development and urbanization [1, 2]. Adoption of healthy lifestyles or behaviors (e.g., no smoking and physical activity) and optimal controls of major biological or cardiometabolic risk factors (e.g., high blood pressure, high cholesterol, and high blood glucose) are well-established strategies to prevent CVD [3, 4].

In 2010, the American Heart Association (AHA) proposed composite cardiovascular health (CVH) metrics that integrated seven lifestyle-related factors (Life’s Simple 7), i.e., smoking, body mass index (BMI), diet, physical activity, blood pressure, blood glucose, and total cholesterol [5]. Systematic reviews suggested that ideal CVH metrics were associated with a reduced risk of cardiovascular events and mortality [6–8]. However, the prevalence of ideal CVH metrics was extremely low in middle-aged and elderly people in many countries, including China [9–13]. In addition, the sex differences in CVH metrics have been previously reported in studies from Europe, whereby ideal CVH metrics were more common in women than in men [14, 15]. However, data from community-based studies that focus on sex disparities in CVH metrics are limited in China, especially among rural-dwelling Chinese older adults.

In the past five decades, the management and control of biological or cardiometabolic components of CVH metrics such as high blood pressure, high cholesterol, and high blood glucose in high-income countries have been steadily improved, [16] which significantly contributes to the declining trends in incidence and mortality of CVD [6]. However, the age-standardized prevalence of CVD had increased from 1990 to 2016 in China [2]. Several studies have reported relatively low rates of medical treatment and effective control of hypertension, diabetes, and high cholesterol among Chinese adult populations [17–19]. However, sex differences in the management of major biological CVH metrics in rural-dwelling older adults remain unclear.

Thus, in the current study, we sought to investigate the sex-specific distribution of CVH metrics and management of biological CVH components among Chinese older adults living in the rural communities in western Shandong province.

## Methods

### Study participants

This is a population-based cross-sectional study. The study sample was derived from participants in the baseline survey of the ongoing Multimodal Interventions to delay Dementia and disability in rural China (MIND-CHINA), which targets people who were aged ≥65 years and living in the rural communities of Yanlou Town, Yanggu County, western Shandong Province, China. In March–September 2018, 5246 participants were examined as part of the baseline survey for MIND-CHINA. Of these, 220 (4.19%) were excluded due to missing data on CVH measurements, leaving 5026 persons for the current analysis.

The Ethics Committee of Shandong Provincial Hospital affiliated to Shandong University in Jinan, China reviewed and approved the protocol for MIND-CHINA. Written informed consent was obtained from all the participants, or in the case of cognitively impaired persons, from a proxy (usually a family member). MIND-CHINA was registered in the Chinese Clinical Trial Registry (registration no.: ChiCTR1800017758).

### Data collection and assessments

The trained staff collected data via face-to-face interviews, clinical examinations, and laboratory tests following a structured questionnaire, which was developed and adapted from questionnaires used in the Study on Global Ageing and Adult Health (SAGE) and a local survey of aging and health [20, 21]. Data included demographic features (e.g., age, sex, and education), lifestyles (e.g., smoking, BMI, and physical activity), health history (e.g., hypertension, diabetes, and CVD), and use of medications in the last two weeks before the survey. Weight and height were measured with participants wearing light clothes and without shoes. BMI was calculated as weight (kg) divided by height squared (m^2^). After a 5-min rest, arterial blood pressure was measured on the right upper arm in a seated position using an electronic blood pressure monitor (HEM-7127 J, Omron Corporation, Kyoto, Japan). We assessed physical activity via questions of frequency (e.g., daily, weekly, and monthly) and time (minutes) of walking, sports activities, and recreational activities. The frequency of physical activity was coded as 7, 1, and 1/4 for daily, weekly, and monthly activity, respectively, and physical activity was quantified as minutes spent per week by multiplying participation frequency with average minutes spent per time. Then we calculated the metabolic-equivalent of each activity according to the 2011 compendium of physical activities [22]. Peripheral blood samples were taken after an overnight fast, and blood samples were analyzed at the certified clinical laboratory of the local town health center. Fasting blood glucose (FBG) and total cholesterol (TC) were measured using an automatic biochemical analyzer (DIRUI CS-600B; DIRUI Corporation, Changchun, China).

Hypertension was defined as systolic pressure ≥ 140 mmHg or diastolic pressure ≥ 90 mmHg or current use of antihypertensive medication, [17] and high cholesterol as TC ≥6.22 mmol/L or having received treatment for high cholesterol, [18] diabetes as self-reported history of diabetes diagnosed by a physician or FBG ≥7.0 mmol/L or current use of blood glucose-lowering medication [19]. “Awareness” of a disease was referred to a self-reported physician diagnosis of the disease before the examination [17]. “Treatment” was referred to self-reported use of medications for a certain disease, and the treatment rate as the proportion of persons who were taking medications among people with the disease [19]. The control rate of hypertension, diabetes, and high cholesterol was defined as the proportion of achieving the goal of blood pressure < 140/90 mmHg, [17] TC < 6.22 mmol/L, [18] and FBG < 7.0 mmol/L, [19] respectively, among people who took the corresponding medications (control A) or among people who had the corresponding disease (control B).

### Definition of cardiovascular health metrics

We defined CVH metrics following the AHA’s recommendations, [5] with some modifications (Supplemental Table 1): (1) we did not include diet due to a lack of dietary data; (2) we defined ideal BMI as < 24 kg/m^2^, as recommended for Chinese adults [23]; and (3) we defined ideal smoking level as never or quitting smoking > 5 years [24]. Thus, we included six factors in the CVH metrics: smoking, BMI, physical activity, blood pressure, total cholesterol, and FBG. We considered smoking, BMI, and physical activity as behavioral CVH metrics, and blood pressure, total cholesterol, and FBG as biological CVH metrics, as previously suggested [5, 24, 25]. We categorized each of the six factors into three levels of poor, intermediate, and ideal. Participants with 0–2, 3–4, and 5–6 metrics at the ideal levels were defined as having poor, intermediate, and ideal global CVH metrics, respectively [5]. Furthermore, people with 0–1, 2, and 3 behavioral or biological CVH metrics at the ideal level were defined as having poor, intermediate, and ideal behavioral or biological CVH metrics, respectively [14].

### Statistical analysis

Descriptive analysis was performed to report mean (standard deviation, SD) for continuous variables with normal distribution, and frequency (proportion) for categorical variables. Characteristics of the study participants by sex were compared using the chi-square test for categorical and t-test for normal distributed continuous variables. We reported the prevalence of individual and composite CVH metrics and the rates of awareness, treatment, and control of biological CVH components. Stata Statistical Software: Release 14 for Windows (StataCorp LLC., College Station, TX, USA) was used for all analyses. Two-tailed *p* < 0.05 was considered to be statistically significant.

## Results

The mean age of the 5026 participants was 71.5 years (SD, 5.3), and 57.2% were women. Compared with men, women were slightly older, less educated (*p* < 0.001), and had a higher level of BMI, systolic pressure, FBG, and TC, but a lower level of diastolic pressure (*p* < 0.001) (Table 1). In addition, only 0.8% of all participants achieved the ideal level in all six CVH metric components.

**Table 1:** Characteristics of study participants by sex

Characteristics	Total sample	Men	Women	*p*-value^a^
No. of participants (%)	5026 (100)	2150 (42.8)	2876 (57.2)	
Age (years)	71.5 (5.3)	71.4 (5.1)	71.6 (5.4)	0.108
Age (years), n (%)				0.001
65–69	2152 (42.8)	939 (43.7)	1213 (42.2)	
70–74	1644 (32.7)	684 (31.8)	960 (33.4)	
75–79	756 (15.0)	356 (16.6)	400 (13.9)	
≥ 80	474 (9.4)	171 (8.0)	303 (10.5)	
Education, n (%)				< 0.001
Illiterate	1992 (39.6)	284 (13.2)	1708 (59.4)	
Primary school	2215 (44.1)	1173 (54.6)	1042 (36.2)	
Middle school or above	819 (16.3)	693 (32.2)	126 (4.4)	
Systolic pressure (mm Hg)	144.1 (21.5)	142.4 (21.2)	145.3 (21.6)	< 0.001
Diastolic pressure (mm Hg)	85.0 (11.0)	85.9 (10.9)	84.3 (10.9)	< 0.001
Fasting blood glucose (mmol/l)	5.6 (1.4)	5.5 (1.3)	5.6 (1.5)	0.001
Total cholesterol (mmol/l)	5.0 (1.0)	4.7 (0.9)	5.2 (1.0)	< 0.001
Body mass index (kg/m^2^)	24.9 (3.8)	24.4 (3.7)	25.2 (3.9)	< 0.001
No. of ideal CVH metric components, n (%)				< 0.001
0	44 (0.9)	43 (2.0)	1 (0.03)	
1	356 (7.1)	194 (9.0)	162 (5.6)	
2	1198 (23.8)	523 (24.3)	675 (23.5)	
3	1739 (34.6)	727 (33.8)	1012 (35.2)	
4	1243 (24.7)	495 (23.0)	748 (26.0)	
5	408 (8.1)	149 (6.9)	259 (9.0)	
6	38 (0.8)	19 (0.9)	19 (0.7)	

Data are mean (standard deviation), unless otherwise specified

Abbreviation: CVH, cardiovascular health

^a^*p*-value is for the test of differences between men and women

Table 2 shows the overall and sex-specific prevalence of individual CVH metrics and composite measures of global, behavioral, and biological CVH metrics. Overall, the prevalence of ideal level in individual CVH metrics ranged from 42.4 to 72.1%, except blood pressure, where only 7.9% achieved an optimal level. The prevalence of ideal level in the composite CVH metrics ranged from 8.9% in global CVH metrics and 4.3% in biological CVH metrics to 14.5% in behavioral CVH metrics. There were significant sex differences in all individual and composite CVH metrics (Table 2). Compared to women, men were more likely to have an ideal level for behavioral CVH metric components, except smoking, where women had an extremely low smoking rate (1.4% in women vs. 46.8% in men, *p* < 0.001). In addition, men had a higher proportion of ideal biological CVH metric components (i.e., TC, FBG, and blood pressure) than women (*p* < 0.05). Furthermore, men had a higher proportion of ideal composite biological CVH metrics (*p* < 0.001), whereas women were more likely to have an ideal level for the composite behavioral CVH metrics (*p* < 0.001).

**Table 2:** Prevalence of cardiovascular health metrics by sex

CVH metrics		Total sample (*n* = 5026)	Men (*n* = 2150)	Women (*n* = 2876)	*p*-value^a^
Smoking status, n (%)	Poor	1045 (20.8)	1006 (46.8)	39 (1.4)	< 0.001
	Intermediate	355 (7.1)	344 (16.0)	11 (0.4)	
	Ideal	3626 (72.1)	800 (37.2)	2826 (98.3)	
Physical activity, n (%)	Poor	1774 (35.3)	708 (32.9)	1066 (37.1)	< 0.001
	Intermediate	612 (12.2)	222 (10.3)	390 (13.6)	
	Ideal	2640 (52.5)	1220 (56.7)	1420 (49.4)	
Body mass index, n (%)	Poor	974 (19.4)	341 (15.9)	633 (22.0)	< 0.001
	Intermediate	1920 (38.2)	792 (36.8)	1128 (39.2)	
	Ideal	2132 (42.4)	1017 (47.3)	1115 (38.8)	
Blood pressure, n (%)	Poor	3148 (62.6)	1295 (60.2)	1853 (64.4)	0.008
	Intermediate	1481 (29.5)	668 (31.1)	813 (28.3)	
	Ideal	397 (7.9)	187 (8.7)	210 (7.3)	
Total cholesterol, n (%)	Poor	513 (10.2)	100 (4.7)	413 (14.4)	< 0.001
	Intermediate	1537 (30.6)	525 (24.4)	1012 (35.2)	
	Ideal	2976 (59.2)	1525 (70.9)	1451 (50.5)	
Fasting blood glucose, n (%)	Poor	437 (8.7)	168 (7.8)	269 (9.4)	0.032
	Intermediate	1151 (22.9)	471 (21.9)	680 (23.6)	
	Ideal	3438 (68.4)	1511 (70.3)	1927 (67.0)	
Global CVH metrics, n (%)	Poor	1598 (31.8)	760 (35.3)	838 (29.1)	< 0.001
	Intermediate	2982 (59.3)	1222 (56.8)	1760 (61.2)	
	Ideal	446 (8.9)	168 (7.8)	278 (9.7)	
Behavioral CVH metrics, n (%)	Poor	2087 (41.5)	1186 (55.2)	901 (31.3)	< 0.001
	Intermediate	2208 (43.9)	760 (35.3)	1448 (50.3)	
	Ideal	731 (14.5)	204 (9.5)	527 (18.3)	
Biological CVH metrics, n (%)	Poor	2740 (54.5)	975 (45.3)	1765 (61.4)	< 0.001
	Intermediate	2069 (41.2)	1058 (49.2)	1011 (35.2)	
	Ideal	217 (4.3)	117 (5.4)	100 (3.5)	

Abbreviation: CVH, cardiovascular health

^a^*p*-value is for the test of differences between men and women

**Fig. 1:**
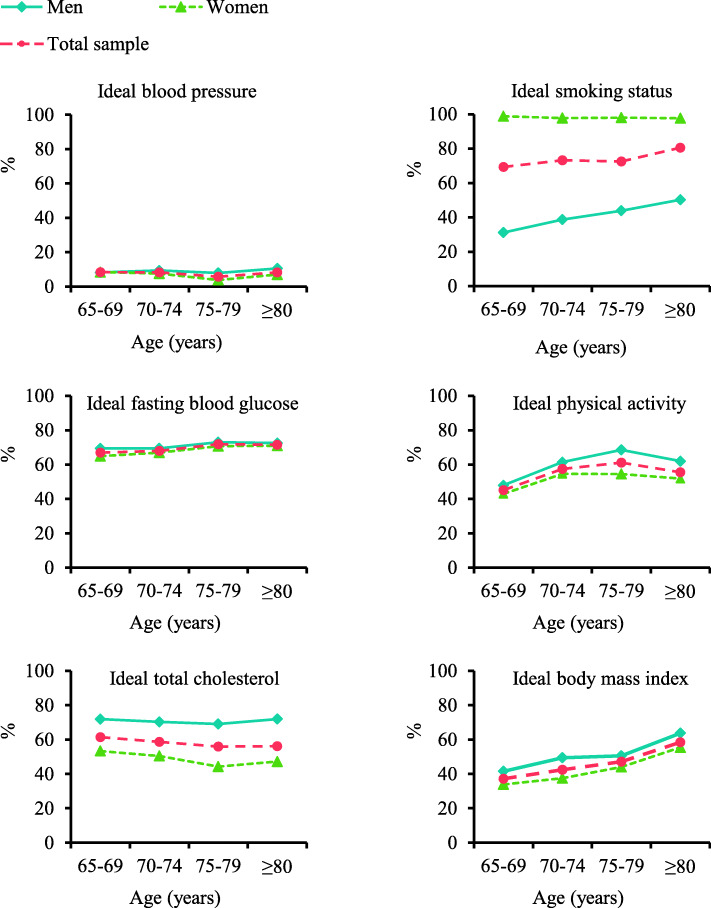
Age- and sex-specific prevalence of ideal individual cardiovascular health metrics (*n* = 5026)

**Fig. 2:**
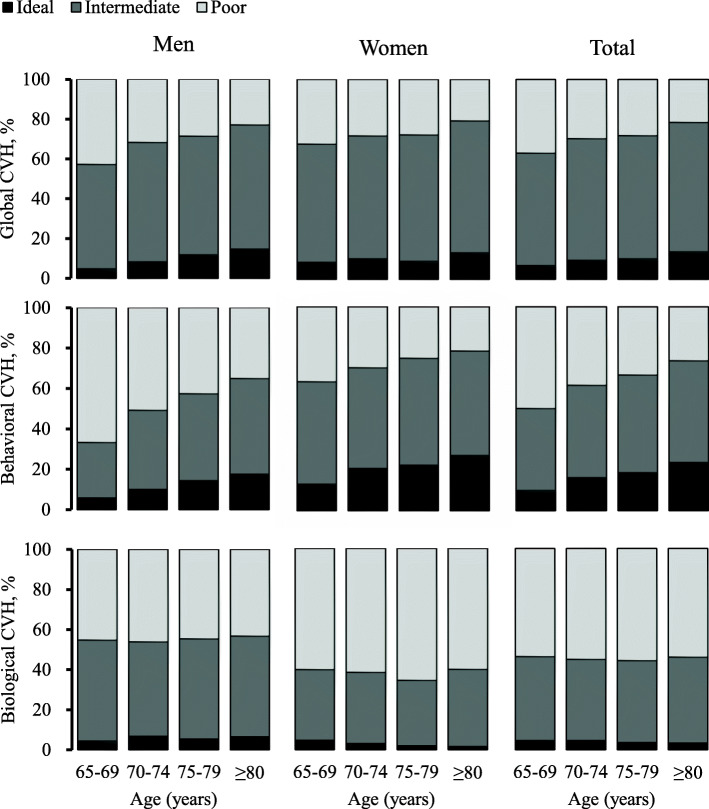
Age- and sex-specific proportions of ideal, intermediate, and poor levels of composite cardiovascular health (CVH) metrics (*n* = 5026)

The prevalence of ideal individual CVH metrics increased with age for BMI (*p* for trend< 0.001), but was stable with age for blood pressure at a very low rate (< 10%) (Fig. 1). The prevalence of ideal FBG and physical activity slightly increased initially until age 75–79, then dropped significantly with age, whereas the prevalence of ideal TC decreased with age until age 75–79, then increased significantly with age (Fig. 1). Moreover, the prevalence of ideal composite global and behavioral CVH metrics increased with age in both women and men (*p* for trend< 0.001), whereas the prevalence of ideal composite biological CVH metrics was stable with age at a relatively low rate (< 10%) (Fig. 2).

We examined the awareness, medical treatment, and control rates of three biological CVH components overall and by sex (Table 3). The overall prevalence of hypertension, diabetes, and high cholesterol was 67.1, 14.4, and 15.1%, respectively. Women had a higher prevalence of hypertension (*p* = 0.001), diabetes (*p* < 0.001), and high cholesterol (*p* < 0.001) than men. Among people with hypertension, 59.4% were aware of their hypertension, and 33.0% were treated with antihypertensive drugs, but only 20.3% of those treated achieved the goal of blood pressure control. Of those with diabetes, 80.7% were aware of their diagnosis, and 33.2% received pharmacological treatment; among those who were treated with blood glucose drugs, only 37.1% achieved the goal of their blood glucose. Among people with high cholesterol, 25.4% were aware of their high cholesterol condition, 34.4% were treated with cholesterol-lowering drugs, and 94.7% of those treated people achieved good control of high total cholesterol. Moreover, the overall control rates among people with the disease were 6.7% for hypertension, 39.5% for diabetes, and 32.6% for high cholesterol. Furthermore, women had higher rates of awareness (*p* < 0.001) and treatment (*p* = 0.016) for hypertension, and higher rates of awareness (*p* < 0.001) and effective control (*p* = 0.003) for diabetes, while men had higher rates of treatment (*p* < 0.001) and effective control (*p* < 0.001) for high cholesterol (Table 3).

**Table 3:** Prevalence and awareness, treatment, and control proportions of biological cardiovascular health metrics in total sample and by sex (*n* = 5026)

Cardiovascular health metrics	n	Proportion (95% confidence interval) (%)			*p*-value^a^
		Total sample (*n* = 5026)	Men (*n* = 2150)	Women (*n* = 2876)	
Hypertension					
Prevalence	3374	67.1 (65.8–68.4)	64.6 (62.5–66.6)	69.1 (67.4–70.7)	0.001
Awareness	2003	59.4 (57.7–61.0)	54.3 (51.7–57.0)	62.9 (60.8–65.0)	< 0.001
Treatment	1113	33.0 (31.4–34.6)	30.8 (28.3–33.2)	34.5 (32.5–36.6)	0.022
Control A^b^	226	20.3 (17.9–22.7)	21.8 (17.9–25.7)	19.4 (16.4–22.4)	0.335
Control B^b^	226	6.7 (5.9–7.5)	6.7 (5.4–8.0)	6.7 (5.6–7.8)	0.991
Diabetes					
Prevalence	722	14.4 (13.4–15.3)	11.6 (10.2–12.9)	16.5 (15.1–17.8)	< 0.001
Awareness	583	80.7 (77.9–83.6)	72.3 (66.7–77.9)	85.2 (82.0–88.4)	< 0.001
Treatment	240	33.2 (29.8–36.7)	32.1 (26.3–38.0)	33.8 (29.6–38.1)	0.645
Control A^b^	89	37.1 (30.9–43.2)	33.8 (21.2–44.3)	38.8 (31.1–46.4)	0.450
Control B^b^	285	39.5 (35.9–43.1)	32.5 (26.7–38.4)	43.1 (38.7–47.6)	0.006
High cholesterol					
Prevalence	761	15.1 (14.2–16.1)	9.8 (8.5–11.0)	19.2 (17.7–20.6)	< 0.001
Awareness	193	25.4 (22.3–28.5)	27.1 (21.1–33.2)	24.7 (21.1–28.3)	0.486
Treatment	262	34.4 (31.1–37.8)	54.8 (48.0–61.6)	26.7 (23.0–30.4)	< 0.001
Control A^b^	248	94.7 (91.9–97.4)	95.7 (91.9–99.4)	93.9 (90.0–97.8)	0.526
Control B^b^	248	32.6 (29.3–35.9)	52.4 (45.6–59.2)	25.1 (21.4–28.7)	< 0.001

^a^*p*-value is for the test of differences between men and women

^b^Control A indicates the proportion of achieving the treatment goal among people who were treated with the corresponding medication; Control B means the proportion of achieving the treatment goal among people who had the corresponding biological cardiovascular risk factor

## Discussion

This large-scale community-based study revealed an overall very low prevalence of ideal global composite CVH metrics (8.9%) as well as substantial sex differences in CVH metrics, such that men were more likely than women to have ideal individual CVH metrics in all but smoking and ideal composite biological CVH metrics, whereas women were more likely to have ideal composite behavioral and global CVH metrics than men. Furthermore, women had higher proportions of awareness of hypertension and diabetes, whereas men had higher proportions of treatment and effective control in high cholesterol. These findings may have implications for precision management of cardiovascular health risks among rural-dwelling older adults.

The extremely low proportion (< 1%) of older adults that could achieve the ideal level in all the examined six components of CVH metrics in our study sample is consistent with several reports from other countries [6, 26–29]. However, the distribution of individual CVH metrics in our study population appears to differ from that of studies from the western societies. The prevalence of ideal BMI, FBG, TC, and physical activity (ranging from 42.4 to 68.4%) were slightly higher than that from western countries, [14, 30] probably because of the favorable traditional lifestyles (except for smoking in men) in the local rural areas, such as the diet of more grain, more vegetables, and less meat consumptions [31]. Unfortunately, we did not have detailed dietary data to compare with the literature. However, the prevalence of ideal blood pressure in our study was very low (< 10%), which could be partially due to the fact that salt intake in northern China (e.g., Shandong Province) is among the highest in the world [32]. Local residents had the diet habit with high sodium foods (e.g., pickles or salted vegetables), especially in the winter, which could partly contribute to high blood pressure, and thus, a relatively high prevalence of hypertension. Moreover, the overall prevalence of current smoking (20.8%) in our study participants was higher than that in western countries (ranging from 10.5 to 16.3%), [14, 30, 33] but the sex difference in smoking habits in our population (46.8% vs. 1.4%) was more substantial compared with reports from western societies.

Given that unhealthy lifestyles and behaviors may result in an unfavorable state of biological or metabolic CVH metrics, it could be expected that the prevalence of ideal composite biological CVH was also very low in our study population (< 5%). Furthermore, the frequency of ideal composite biological CVH metrics was lower than that of composite behavioral CVH metrics (4.3% vs. 14.5%), which is consistent with previous studies [14]. This suggests that biological or metabolic risk factors might contribute even more heavily than unhealthy lifestyles or behavioral factors to poor cardiovascular health, which highlights the need to promote healthy behaviors and improve the management of cardiometabolic risk factors in rural China. It is worth noting that several well-designed studies have classified BMI as a component of behavioral CVH metrics following the AHA’s recommendations, [14, 25, 34, 35] although it has been argued that obesity should be regarded as a multifactorial complex disease [36].

The sex differences in composite measurements of CVH metrics have been rarely explored in the literature. We found that women were more likely to have ideal composite global and behavioral CVH metrics, which is in accordance with the previous cross-sectional studies of middle-aged and elderly people in France [6, 14]. Of note, the sex differences in global and behavioral CVH metrics in our study might be driven primarily by smoking habits, where the prevalence of smoking was very high in men (46.8%) but very low in women (1.4%) owing to socio-cultural tradition and social environment in Asia and China, especially in rural regions [37]. Besides, men were more likely to have ideal biological CVH metrics in our study of older adults, which is in contrast to a previous study of middle-aged and older people in France [14]. This discrepancy is likely due to the facts that premenopausal women are relatively protected against cardiovascular and metabolic risks and that the sex differences diminish with advancing age, particularly after menopause [38].

Previous studies have shown that major lifestyles and cardiometabolic risk factors are poorly controlled in low- and middle-income countries, especially among residents living in remote rural areas [20, 33, 39]. Findings from our community-based study are in line with those previous reports and further showed the distinct profiles for these risk factors between men and women. Despite of highly prevalent cardiometabolic risk factors, only around one-third of people with these metabolic risk factors were treated, and even lower proportions of those who were treated achieved the goals of treatment. Notably, we found that the proportion of effective control of diabetes was even slightly higher among people with diabetes than those with diabetes and medical treatment (Table 3). This suggests that some people who reported to have the diagnosis of diabetes actually had their FBG returned to be normal (< 7 mmol/l) but without use of glucose-lowing medicines. A possible explanation is that some people with diabetes might be able to achieve the goal of blood glucose control without taking blood-glucose lowering medications, possibly via adaptation of healthy lifestyles (e.g., diet and physical activity) [40]. This is particularly the case for people with newly diagnosed diabetes whose FBG levels are fluctuating and varying greatly within any given day and from day to day [41]. Moreover, we found that women were more likely than men to be aware of their hypertension and diabetes. As a result, in spite of a higher prevalence of hypertension in women than in men, the rates of achieving the goal of blood pressure control were comparable between men and women. As for high total cholesterol, the proportions of treatment and effective control were higher in men than in women, which were consistent with the reports from a previous study of rural-dwelling older adults in China [20]. Of note, women had a higher prevalence of hypertension, diabetes, and high cholesterol than men, which is consistent with sex difference in biological CVH metrics in our study. Taken together, the biological CVH risk profiles of the rural-dwelling older adults were characterized by highly prevalent and poorly controlled biological or cardiometabolic risk factors. Thus, additional efforts are needed to promote cardiovascular health by improving medical treatment and effective control of these risk factors.

The strengths of this study include the community-based design, the relatively large sample of the rural residents in China, and comprehensive assessments of major CVH metrics. However, our study also has limitations. Firstly, physical activity and smoking status were self-reported, which may be subject to recall bias. Secondly, we were not able to follow the AHA’s original recommendations in defining CVH metrics due to a lack of dietary data. Finally, the study participants from only one rural area may not be representative of the rural population in China, which should be kept in mind when generalizing the study findings to other rural populations.

## Conclusions

This community-based study reveals that the overall prevalence of ideal CVH metrics was very low, along with distinct sex differences in individual and composite CVH metrics, and that the biological or metabolic CVH components were poorly controlled among Chinese rural-dwelling older adults. These results call for more efforts to improve cardiovascular health among rural residents in China. Furthermore, characterizing the sex differences in CVH metrics may help achieve precision preventive interventions to reduce CVD burden among older Chinese people.

## Supplementary Information

**Additional file 1: Supplementary Material S1.** Definitions of cardiovascular health metrics.

## Data Availability

The datasets used and/or analyzed during the current study are available from the corresponding author upon reasonable request.

## Abbreviations

CVH: cardiovascular health
CVD: cardiovascular disease
AHA: American Heart Association
BMI: body mass index
MIND-CHINA: Multimodal Interventions to delay Dementia and disability in rural China
FBG: fasting blood glucose
SAGE: Study on Global Ageing and Adult Health
TC: total cholesterol
SD: standard deviation

## References

[1] Yang G, Wang Y, Zeng Y, Gao GF, Liang X, Zhou M, Wan X, Yu S, Jiang Y, Naghavi M (2013). Rapid health transition in China, 1990-2010: findings from the global burden of disease study 2010. Lancet.

[2] Liu S, Li Y, Zeng X, Wang H, Yin P, Wang L, Liu Y, Liu J, Qi J, Ran S (2019). Burden of cardiovascular diseases in China, 1990-2016: findings from the 2016 global burden of disease study. JAMA Cardiol.

[3] Chiuve SE, McCullough ML, Sacks FM, Rimm EB (2006). Healthy lifestyle factors in the primary prevention of coronary heart disease among men: benefits among users and nonusers of lipid-lowering and antihypertensive medications. Circulation.

[4] Hardoon SL, Whincup PH, Lennon LT, Wannamethee SG, Capewell S, Morris RW (2008). How much of the recent decline in the incidence of myocardial infarction in British men can be explained by changes in cardiovascular risk factors? Evidence from a prospective population-based study. Circulation.

[5] Lloyd-Jones DM, Hong Y, Labarthe D, Mozaffarian D, Appel LJ, Van Horn L, Greenlund K, Daniels S, Nichol G, Tomaselli GF (2010). Defining and setting national goals for cardiovascular health promotion and disease reduction: the American Heart Association's strategic impact goal through 2020 and beyond. Circulation.

[6] Gaye B, Canonico M, Perier MC, Samieri C, Berr C, Dartigues JF, Tzourio C, Elbaz A, Empana JP (2017). Ideal cardiovascular health, mortality, and vascular events in elderly subjects: the Three-City study. J Am Coll Cardiol.

[7] Guo L, Zhang S (2017). Association between ideal cardiovascular health metrics and risk of cardiovascular events or mortality: a meta-analysis of prospective studies. Clin Cardiol.

[8] Zhou L, Zhao L, Wu Y, Wu Y, Gao X, Li Y, Mai J, Nie Z, Ou Y, Guo M (2018). Ideal cardiovascular health metrics and its association with 20-year cardiovascular morbidity and mortality in a Chinese population. J Epidemiol Community Health.

[9] Ren J, Guo XL, Lu ZL, Zhang JY, Tang JL, Chen X, Gao CC, Xu CX, Xu AQ (2016). Ideal cardiovascular health status and its association with socioeconomic factors in Chinese adults in Shandong, China. BMC Public Health.

[10] Bi Y, Jiang Y, He J, Xu Y, Wang L, Xu M, Zhang M, Li Y, Wang T, Dai M (2015). Status of cardiovascular health in Chinese adults. J Am Coll Cardiol.

[11] Benziger CP, Zavala-Loayza JA, Bernabe-Ortiz A, Gilman RH, Checkley W, Smeeth L, Malaga G, Miranda JJ, group CCS (2018). Low prevalence of ideal cardiovascular health in Peru. Heart.

[12] Peng Y, Cao S, Yao Z, Wang Z (2018). Prevalence of the cardiovascular health status in adults: a systematic review and meta-analysis. Nutr Metab Cardiovasc Dis.

[13] Younus A, Aneni EC, Spatz ES, Osondu CU, Shaharyar S, Roberson L, Ali SS, Ogunmoroti O, Ahmad R, Post J (2015). Prevalence of ideal cardiovascular health among adults in the United States. J Am Coll Cardiol.

[14] Simon M, Boutouyrie P, Narayanan K, Gaye B, Tafflet M, Thomas F, Guibout C, Périer MC, Pannier B, Jouven X (2017). Sex disparities in ideal cardiovascular health. Heart.

[15] Janković J, Marinković J, Stojisavljević D, Erić M, Vasiljević N, Janković S (2016). Sex inequalities in cardiovascular health: a cross-sectional study. Eur J Pub Health.

[16] Peters SAE, Muntner P, Woodward M (2019). Sex differences in the prevalence of, and trends in, cardiovascular risk factors, treatment, and control in the United States, 2001 to 2016. Circulation.

[17] Lu J, Lu Y, Wang X, Li X, Linderman GC, Wu C, Cheng X, Mu L, Zhang H, Liu J (2017). Prevalence, awareness, treatment, and control of hypertension in China: data from 1·7 million adults in a population-based screening study (China PEACE million persons project). Lancet.

[18] Song P, Zha M, Yang X, Xu Y, Wang H, Fang Z, Yang X, Xia W, Zeng C (2019). Socioeconomic and geographic variations in the prevalence, awareness, treatment and control of dyslipidemia in middle-aged and older Chinese. Atherosclerosis.

[19] Liu X, Li Y, Li L, Zhang L, Ren Y, Zhou H, Cui L, Mao Z, Hu D, Wang C (2016). Prevalence, awareness, treatment, control of type 2 diabetes mellitus and risk factors in Chinese rural population: the RuralDiab study. Sci Rep.

[20] Song A, Liang Y, Yan Z, Sun B, Cai C, Jiang H, Qiu C (2014). Highly prevalent and poorly controlled cardiovascular risk factors among Chinese elderly people living in the rural community. Eur J Prev Cardiol.

[21] Kowal P, Chatterji S, Naidoo N, Biritwum R, Fan W, Lopez Ridaura R, Maximova T, Arokiasamy P, Phaswana-Mafuya N, Williams S (2012). Data resource profile: the World Health Organization study on global AGEing and adult health (SAGE). Int J Epidemiol.

[22] Ainsworth BE, Haskell WL, Herrmann SD, Meckes N, Bassett DR, Tudor-Locke C, Greer JL, Vezina J, Whitt-Glover MC, Leon AS (2011). 2011 compendium of physical activities: a second update of codes and MET values. Med Sci Sports Exerc.

[23] Zhou BF (2002). Predictive values of body mass index and waist circumference for risk factors of certain related diseases in Chinese adults--study on optimal cut-off points of body mass index and waist circumference in Chinese adults. Biomed Environ Sci.

[24] Sabia S, Fayosse A, Dumurgier J, Schnitzler A, Empana JP, Ebmeier KP, Dugravot A, Kivimäki M, Singh-Manoux A (2019). Association of ideal cardiovascular health at age 50 with incidence of dementia: 25 year follow-up of Whitehall II cohort study. BMJ.

[25] Liang Y, Ngandu T, Laatikainen T, Soininen H, Tuomilehto J, Kivipelto M, Qiu C (2020). Cardiovascular health metrics from mid- to late-life and risk of dementia: a population-based cohort study in Finland. PLoS Med.

[26] Garcia-Hermoso A, Ramirez-Velez R, Ramirez-Campillo R, Izquierdo M (2018). Prevalence of ideal cardiovascular health and its association with cognitive function in older adults: the Chilean National Health Survey (2009-2010). Rejuvenation Res.

[27] Graciani A, García-Esquinas E, López-García E, Banegas JR, Rodríguez-Artalejo F (2016). Ideal cardiovascular health and risk of frailty in older adults. Circ Cardiovasc Qual Outcomes.

[28] Sturlaugsdottir R, Aspelund T, Bjornsdottir G, Sigurdsson S, Eiriksdottir G, Imai CM, Garcia M, Launer LJ, Harris TB, Gudnason V (2015). Carotid atherosclerosis and cardiovascular health metrics in old subjects from the AGES-Reykjavik study. Atherosclerosis.

[29] Samieri C, Perier MC, Gaye B, Proust-Lima C, Helmer C, Dartigues JF, Berr C, Tzourio C, Empana JP (2018). Association of Cardiovascular Health Level in older age with cognitive decline and incident dementia. JAMA.

[30] Jin Y, Tanaka T, Bandinelli S, Ferrucci L, Talegawkar SA (2017). Overall cardiovascular health is associated with all-cause and cardiovascular disease mortality among older community-dwelling men and women. J Aging Health.

[31] Chang Y, Guo X, Chen Y, Guo L, Li Z, Yu S, Yang H, Sun G, Sun Y (2016). Prevalence and metrics distribution of ideal cardiovascular health: a population-based, cross-sectional study in rural China. Heart Lung Circ.

[32] Tan M, He FJ, Wang C, MacGregor GA (2019). Twenty-four-hour urinary sodium and potassium excretion in China: a systematic review and meta-analysis. J Am Heart Assoc.

[33] Bambs C, Kip KE, Dinga A, Mulukutla SR, Aiyer AN, Reis SE (2011). Low prevalence of "ideal cardiovascular health" in a community-based population: the heart strategies concentrating on risk evaluation (heart SCORE) study. Circulation.

[34] Rangé HPM, Boillot A, Offredo L, Lisan Q, Guibout C, Thomas F, Danchin N, Boutouyrie P, Jouven X, Bouchard P, Empana JP (2020). Chewing capacity and ideal cardiovascular health in adulthood: a cross-sectional analysis of a population-based cohort study. Clin Nutr.

[35] Mazidi MKN, Mikhailidis DP, Banach M (2019). Ideal cardiovascular health associated with fatty liver: results from a multi-ethnic survey. Atherosclerosis.

[36] Gordon-Larsen P, Heymsfield SB (2018). Obesity as a disease, not a behavior. Circulation.

[37] Liu S, Zhang M, Yang L, Li Y, Wang L, Huang Z, Wang L, Chen Z, Zhou M (2017). Prevalence and patterns of tobacco smoking among Chinese adult men and women: findings of the 2010 national smoking survey. J Epidemiol Community Health.

[38] Sanghavi M, Gulati M (2015). Sex differences in the pathophysiology, treatment, and outcomes in IHD. Curr Atheroscler Rep.

[39] Song P, Rudan D, Wang M, Chang X, Rudan I (2019). National and subnational estimation of the prevalence of peripheral artery disease (PAD) in China: a systematic review and meta-analysis. J Glob Health.

[40] Kolb H, Martin S (2017). Environmental/lifestyle factors in the pathogenesis and prevention of type 2 diabetes. BMC Med.

[41] Mellitus. TECotDaCoD (2003). Follow-up report on the diagnosis of diabetes mellitus. Diabetes Care.

