# Supplementary material for: Sex disparities in cardiovascular health metrics among rural-dwelling older adults in China: a population-based study
Source: BMC Geriatr. 2021 Mar 4;21:158. doi: 10.1186/s12877-021-02116-x (PMC7934439; doi:10.1186/s12877-021-02116-x)
Supplement: Supplementary file 1 — Additional file 1: Supplementary Material S1. Definitions of cardiovascular health metrics. [file 12877_2021_2116_MOESM1_ESM.docx]

**Supplemental Table**

**Supplemental Table 1. Definitions of cardiovascular health metrics**

| **Cardiovascular health metrics** | **Ideal level** | **Intermediate level** | **Poor level** |
| --- | --- | --- | --- |
| **Smoking** | Never or quit smoking >5 years | Former smoking and quit smoking ≤5 years | Current |
| **Physical activity** | ≥150 min/week of moderate intensity  or ≥75 min/week of vigorous intensity  or ≥150 min /week moderate + vigorous | 1-149 min/week of moderate intensity  or 1-74 min/week of vigorous intensity  or 1-149 min/week of moderate + vigorous | None or no regular physical activity |
| **Body mass index** | <24 kg/m^2^ | 24-28 kg/m^2^ | ≥28 kg/m^2^ |
| **Blood pressure** | <120/80 mm Hg, untreated | 120-139/80-89 mm Hg or treated to <120/80 mm Hg | ≥140/90 mm Hg |
| **Total cholesterol** | <5.26 mmol/L, untreated | 5.26-6.22 mmol/L  or treated to <5.26 mmol/L | ≥6.22 mmol/L |
| **Fasting blood glucose** | <5.60 mmol/L, untreated | 5.60-6.90 mmol/L  or treated to <5.60 mmol/L | ≥7.00 mmol/L |

Source: Lloyd-Jones DM, et al., *Circulation.* 2010;121(4):586-613 (with modifications).
